# Uptake of Maternal RSV Vaccination and Infant Nirsevimab Among Infants Born October 2023 to March 2024

**DOI:** 10.1001/jamanetworkopen.2024.53696

**Published:** 2025-01-08

**Authors:** Karen B. Jacobson, Andrew J. Watson, Maqdooda Merchant, Bruce Fireman, Ousseny Zerbo, Nicola P. Klein

**Affiliations:** 1 Vaccine Study Center, Division of Research, Kaiser Permanente Northern California, Oakland

## Abstract

This cohort study examines uptake of maternal respiratory syncytial virus (RSV) vaccination and infant receipt of nirsevimab during the early period of availability for these medications.

## Introduction

In 2023, 2 new measures to protect infants against respiratory syncytial virus (RSV) became available: a vaccine (RSVpreF) for pregnant persons at 32 to 36 weeks’ gestation and a monoclonal antibody (nirsevimab) for infants aged younger than 8 months.^1,2,3^ Despite nationwide nirsevimab shortages, both nirsevimab and RSVpreF were widely available soon after approval within Kaiser Permanente Northern California (KPNC), an integrated health care delivery organization. Uptake of maternal vaccines against respiratory pathogens is lower among younger individuals and Black or Hispanic individuals.^4^ We investigated uptake of RSVpreF and nirsevimab and sociodemographic factors associated with uptake within KPNC during the early period of availability.

## Methods

This cohort study was deemed exempt and granted a waiver of informed consent by the institutional review board of KPNC because this was a data-only study. We followed the Strengthening the Reporting of Observational Studies in Epidemiology (STROBE) reporting guideline.

In this cohort study using electronic medical record data among infants born to mothers age 15 to 49 years at KPNC between October 17, 2023 (when nirsevimab became available; RSVpreF became available October 25), and March 31, 2024, we calculated the percentage of infants receiving nirsevimab, infants whose mothers received RSVpreF, infants exposed to both, and those exposed to neither. We compared maternal characteristics, including race and ethnicity (Asian, Black, Hispanic, White, and other [eg, American Indian or Alaska Native, Native Hawaiian or Pacific Islander]), age, parity, neighborhood deprivation index, insurance type, and prenatal visit frequency, among infants in different categories using χ^2^ and Kruskal-Wallis tests. Race and ethnicity were determined from the electronic medical record and self-reported data and included in analysis due to racial disparities in vaccination previously reported in the literature.^4^

*P* values were 2-sided, and statistical significance was set at *P* ≤ .05. Analyses were conducted using SAS Studio, release 3.81 (Enterprise Edition; SAS Institute) from April to November 2024.

## Results

The study included 17 251 infants (8874 [51.4%] male; mean [SD] maternal age, 31.2 [5.2] years), of whom 13 366 (77.5%) received protection from either RSVpreF or nirsevimab. There were 5855 infants (33.9%) exposed to only maternal RSVpreF vaccine, at median (IQR) gestational age of 34.0 (32.6-36.0) weeks (Table). Median (IQR) time from RSVpreF to delivery was 34 (24-46) days. A total of 7051 infants (40.9%) received only nirsevimab (median [IQR] age, 4 [2-16] days). Furthermore, 460 infants (2.7%) were exposed to both maternal RSVpreF and nirsevimab, of whom 143 (31.1%) were born preterm and 111 (24.1%) were admitted to the neonatal intensive care unit. Of 534 infants born within 2 weeks after maternal RSVpreF, 388 (72.7%) also received nirsevimab.

**Table.** Characteristics of Mothers and Infants Receiving RSVpreF and Nirsevimab, 2023-2024

Characteristic	Total Infants (n = 17 251)	Infants, No. (%)				*P* value
		With only maternal RSVpreF (n = 5855 [33.9%])	With only nirsevimab (n = 7051 [40.9%])	With maternal RSVpreF and nirsevimab (n = 460 [2.7%])	Without either RSVpreF or nirsevimab (n = 3885 [22.5%])	
Maternal race and ethnicity^a^						
Asian	4574	1870 (40.9)	1952 (42.7)	143 (3.1)	609 (13.3)	<.001^b^
Black	1036	263 (25.4)	438 (42.3)	26 (2.5)	309 (29.8)	
Hispanic (any race)	5147	1541 (29.9)	2234 (43.4)	129 (2.5)	1243 (24.1)	
White	5351	1788 (33.4)	1957 (36.6)	129 (2.4)	1477 (27.6)	
Multiple or other^c^	537	179 (33.3)	217 (40.4)	17 (3.2)	124 (23.1)	
Pregnancy onset age, y						
15-24	1814	478 (26.4)	802 (44.2)	40 (2.2)	494 (27.2)	<.001^b^
25-29	4184	1279 (30.6)	1695 (40.5)	98 (2.3)	1112 (26.6)	
30-34	6645	2350 (35.4)	2708 (40.8)	179 (2.7)	1408 (21.2)	
35-49	4608	1748 (37.9)	1846 (40.1)	143 (3.1)	871 (18.9)	
Maternal parity^d^						
1	7807	2941 (37.7)	3186 (40.8)	229 (2.9)	1451 (18.6)	<.001^b^
2	5899	2016 (34.2)	2435 (41.3)	140 (2.4)	1308 (22.2)	
3	2322	628 (27.0)	954 (41.1)	63 (2.7)	677 (29.2)	
≥4	1208	268 (22.2)	468 (38.7)	28 (2.3)	444 (36.8)	
Maternal insurance type^e^						
Commercial	14 753	5210 (35.3)	6022 (40.8)	385 (2.6)	3136 (21.3)	<.001^b^
Noncommercial^b^	2494	644 (25.8)	1027 (41.2)	75 (3.0)	748 (30.0)	
NDI quartile^f^						
First (least deprived)	3786	1605 (42.4)	1475 (39.0)	106 (2.8)	600 (15.8)	<.001^b^
Second	4646	1635 (35.2)	1830 (39.4)	106 (2.3)	1075 (23.1)	
Third	4986	1570 (31.5)	2045 (41.0)	120 (2.4)	1251 (25.1)	
Fourth (most deprived)	3826	1043 (27.3)	1697 (44.4)	128 (3.3)	958 (25.0)	
Prenatal visits during 32 to <37 wk gestation, No.						
0	659	24 (3.6)	398 (60.4)	7 (1.1)	230 (34.9)	<.001^b^
1	3179	778 (24.5)	1512 (47.6)	72 (2.3)	817 (25.7)	
2	8714	3184 (36.5)	3432 (39.4)	186 (2.1)	1912 (21.9)	
3	2352	1043 (44.3)	768 (32.7)	65 (2.8)	476 (20.2)	
≥4	2347	826 (35.2)	941 (40.1)	130 (5.5)	450 (19.2)	
NICU status^g^						
Not admitted	15 703	5532 (35.2)	6236 (39.7)	349 (2.2)	3586 (22.8)	<.001^b^
Admitted	1548	323 (20.9)	815 (52.6)	111 (7.2)	299 (19.3)	
Gestational age at delivery, median (IQR), wk^h^	39.3 (38.3-40.1)	39.3 (38.7-40.1)	39.3 (38.1-40.1)	37.3 (36.6-38.0)	39.3 (38.4-40.1)	<.001^h^
Birth weight, median (IQR), g	3315 (2990-3637)	3330 (3040-3640)	3295 (2950-3630)	2910 (2532.5-3210)	3365 (3030-3690)	<.001^h^
Mother received TDAP during pregnancy^i^	15 015 (87.0)	5718 (97.7)	6328 (89.7)	443 (96.3)	2526 (65.0)	<.001^b^
Maternal influenza vaccine during pregnancy^i^	9668 (56.0)	4441 (75.8)	3990 (56.6)	342 (74.3)	895 (23.0)	<.001^b^
Maternal SARS-CoV-2 vaccine during pregnancy^i^	2926 (17.0)	1772 (30.3)	923 (13.1)	119 (25.9)	112 (2.9)	<.001^b^

Abbreviations: NDI, neighborhood deprivation index; NICU, neonatal intensive care unit; TDAP, tetanus, diphtheria, and pertussis.

^a^ Missing data for 606 individuals (3.5%).

^b^ χ^2^ *P* value.

^c^ Includes American Indian or Alaska Native, Native Hawaiian or Pacific Islander, and multiracial.

^d^ Parity includes the infant born in the 2023 to 2024 respiratory virus season. Missing data for 15 individuals (0.1%).

^e^ Noncommercial indicates Medicaid, Medicare, or other subsidized insurance. Missing data for 4 individuals (<0.1%).

^f^ Missing data for 7 individuals (<0.1%).

^g^ Level 2 or 3 NICU.

^h^ Kruskal-Wallis *P* value.

^i^ Percentage given for column total, not row.

Most infants (3615 of 4851 [74.5%]) born October to November received nirsevimab, while more than half of infants (4817 of 9213 [52.3%]) born January to March were exposed to maternal RSVpreF (Figure).

**Figure.**
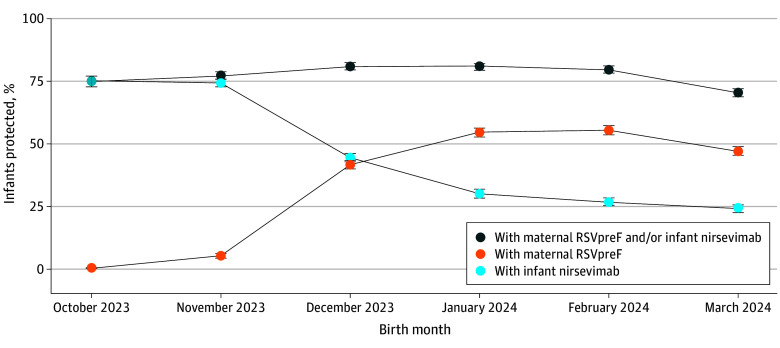
Infants Protected Against Respiratory Syncytial Virus by Birth Month Within Kaiser Permanente Northern California

Uptake of RSVpreF and nirsevimab differed by maternal age and race (Table). Infants of mothers aged younger than 25 years were less likely than infants of mothers aged 35 years or older to be exposed to only RSVpreF (26.4% [95% CI, 24.3%-28.4%] vs 37.9% [95% CI, 36.5%-39.4%]), but more likely to receive only nirsevimab (44.2% [95% CI, 41.9%-46.5%] vs 40.1% [95% CI, 38.6%-41.5%]). Exposure to RSVpreF and/or nirsevimab was highest among infants of Asian mothers (86.7% [95% CI, 85.7%-87.7%]) and lowest among infants of Black mothers (70.2% [95% CI, 67.3%-72.9%]). However, the proportion receiving only nirsevimab was similar among infants of Black (42.3% [95% CI, 39.2%-45.4%]) and Asian (42.7% [95% CI, 41.2%-44.1%]) mothers and lower in infants of White mothers (36.6% [95% CI, 35.3%-37.9%]).

## Discussion

This cohort study in a health care system with ample supply found that nearly 80% of infants born during the 2023 to 2024 RSV season received RSV protection. Most infants born before December received nirsevimab, while most infants of mothers who reached eligible gestational age after October 25 were exposed to maternal RSVpreF. Most nirsevimab administrations occurred in the first week of life, providing early protection. Most infants born within 2 weeks of maternal RSVpreF appropriately received nirsevimab after birth, since they had insufficient time for antibody transfer. Infants receiving both were also more often medically fragile. Infants of younger mothers and Black mothers—historically undervaccinated groups^4^—had lower overall RSV protection coverage, seemingly driven by discrepancies in maternal RSVpreF vaccination more than infant nirsevimab. This study was limited to an insured population and may not be generalizable. The availability of 2 different RSV protection methods in sufficient supply may have contributed to an increased proportion of infants protected against RSV, which may help mitigate disparities.

## Notes

Supplement.
Data Sharing Statement
