# Supplementary material for: Uptake of Maternal RSV Vaccination and Infant Nirsevimab Among Infants Born October 2023 to March 2024
Source: JAMA Netw Open. 2025 Jan 8;8(1):e2453696. doi: 10.1001/jamanetworkopen.2024.53696 (PMC12543396; doi:10.1001/jamanetworkopen.2024.53696)
Supplement: Supplement. — Data Sharing Statement [file jamanetwopen-e2453696-s001.pdf]

Data Sharing Statement

Jacobson. Uptake of Maternal RSV Vaccination and Infant Nirsevimab Among Infants Born October 2023 to March 2024. *JAMA Netw Open*. Published January 08, 2025. doi:10.1001/jamanetworkopen.2024.53696

Data
Data available: No

Additional Information
Explanation for why data not available: The data cannot be shared publicly because the data contain potentially identifying or sensitive patient information and is legally restricted by Kaiser Permanente Northern California. Data are available for researchers who meet the criteria for access to Kaiser Permanente Northern California confidential data. Data requests may be sent to Kaiser Permanente Division of Research: DOR.IRB.Submissions@kp.org
